# Paternal Methyl Donor Supplementation in Rats Improves Fertility, Physiological Outcomes, Gut Microbial Signatures and Epigenetic Markers Altered by High Fat/High Sucrose Diet

**DOI:** 10.3390/ijms22020689

**Published:** 2021-01-12

**Authors:** Faye Chleilat, Alana Schick, Julie M. Deleemans, Raylene A. Reimer

**Affiliations:** 1 Faculty of Kinesiology, University of Calgary, Calgary, AB T2N 1N4, Canada; fatima.chleilat1@ucalgary.ca; 2 International Microbiome Centre, Cumming School of Medicine, University of Calgary, Calgary, AB T2N 4N1, Canada; alana.schick@bcchr.ca; 3 Division of Medical Science, Cumming School of Medicine, University of Calgary, Calgary, AB T2N 4N1, Canada; julie.deleemans@ucalgary.ca; 4 Division of Psychosocial Oncology, Cumming School of Medicine, University of Calgary, Calgary, AB T2N 4N1, Canada; 5 Department of Biochemistry and Molecular Biology, Cumming School of Medicine, University of Calgary, Calgary, AB T2N 4N1, Canada

**Keywords:** paternal nutritional programming, one-carbon metabolism, gut microbiota, DNMT, microRNA, insulin resistance

## Abstract

Increased consumption of high fat/sucrose (HF/S) diets has contributed to rising rates of obesity and its co-morbidities globally, while also negatively impacting male reproductive health. Our objective was to examine whether adding a methyl donor cocktail to paternal HF/S diet (HF/S+M) improves health status in fathers and offspring. From 3–12 weeks of age, male Sprague Dawley rats consumed a HF/S or HF/S+M diet. Offspring were followed until 16 weeks of age. Body composition, metabolic markers, gut microbiota, DNA methyltransferase (DNMT) and microRNA expression were measured in fathers and offspring. Compared to HF/S, paternal HF/S+M diet reduced fat mass in offspring (*p* < 0.005). HF/S+M fathers consumed 16% fewer kcal/day, which persisted in HF/S+M female offspring and was explained in part by changes in serum glucagon-like peptide-1 (GLP-1) and peptide tyrosine tyrosine (PYY) levels. Compared to HF/S, HF/S+M fathers had a 33% improvement in days until conception and 300% fewer stillbirths. In fathers, adipose tissue DNMT3a and hepatic miR-34a expression were reduced with HF/S+M. Adult male offspring showed upregulated miR-24, -33, -122a and -143 expression while females exhibited downregulated miR-33 expression. Fathers and offspring presented differences in gut microbial signatures. Supplementing a paternal HF/S diet with methyl-donors improved fertility, physiological outcomes, epigenetic and gut microbial signatures intergenerationally.

## 1. Introduction

Growing evidence suggests that the decline in male reproductive fitness globally, is in part due to the growing obesity epidemic [1]. Human [2,3] and animal studies [4] have shown that high fat diets, which often reflect poor nutritional status and contribute to increased body mass index (BMI), have been associated with compromised sperm quality, embryo development and fetal growth. Obesity is characterized by excess adipose tissue as well as the deposition of fat in ectopic locations [5], increased plasma fatty acids and an augmented occurrence of insulin resistance [6]. In murine models, high fat diet-induced hyperglycemia, hyperinsulinemia and hypercholesterolemia are associated with alterations in testicular morphology [7,8]. The relationship between male reproductive health and obesity is predictable based on the fact that cholesterol is the chief metabolic precursor involved in testosterone synthesis [9] and the principal spermatogenesis regulator [10], both of which are modulated in part by epigenetics.

Epigenetics refers to modifications to DNA that affect gene expression profiles of a cell but occur in the absence of changes to the DNA sequence [11]. In comparison to the genome, there is considerable plasticity at the cellular epigenetic level. Among the most widely studied epigenetic mechanisms are DNA methylation and non-coding RNA expression [12].

Non-coding RNAs (ncRNAs) are made up of long (>200 nucleotides) or small (<200 nucleotides) ncRNAs, which function as small housekeeping or regulatory ncRNA, the latter including microRNAs [12]. MicroRNAs (19–25 bp in length) function as mRNA translation suppressors or inducers of mRNA breakdown in mammalian cells [13]. In the liver, miRNAs are postulated to coordinate cell lineage differentiation during organ development, playing a crucial role in lipid metabolism and disease modulation, making them valuable prognostic and therapeutic biomarkers clinically [14]. 

DNA methylation is largely associated with gene regulation and cell differentiation. The primary targets of methylation are CpG dinucleotides in non-coding regions, like promotors [12]. DNA methylation involves the careful coordination of methylating enzymes known as DNA methyl transferases (DNMTs). DNMTs transfer a methyl group from S-adenosylmethionine (SAM) to a cytosine residue, ultimately forming 5-methylcytosine [12]. The varying types of DNMTs include DNMT3a and 3b which control de novo DNA methylation activity and DNMT1, which maintains methylation profiles during DNA replication and cell division [15]. A by-product of the methyltransferase reaction is homocysteine (Hcy) [16]. DNA methylation machinery operate on a myriad of different tissues, most notably, adipocytes, which modulate important adipocyte metabolic functions [17]. Moreover, emerging evidence has postulated that DNA methylation is involved in the transgenerational inheritance of obesity and metabolic syndrome [18]. 

One carbon metabolism is comprised of the interconnected folate and methionine cycles that facilitate the transfer of 1C moieties required for cellular processes [19]. The key dietary constituents that mediate one carbon metabolism are folate, other B vitamins (B2, B6, and B12), methionine, choline and betaine. Amino acid methionine levels in the body are dependent on dietary intake, protein catabolism and the re-methylation of Hcy [20]. Methionine can be converted into SAM, which functions as a universal methyl donor in most methyltransferase reactions [21]. Vitamin B12 is an important co-factor for methionine synthase, the rate-limiting enzyme that converts Hcy to methionine [22]. It is via Hcy remethylation that the folate cycle and methionine cycle are linked [20]. The primary role of folate is to donate or accept one-carbon units, which it primarily does via tetrahydrofolate [23]. Betaine, which is derived from the oxidation of choline, can also be used as a methyl donor to recycle Hcy to methionine [24]. The availability of methyl groups originating from diet (derived from methyl-folate, methionine or choline/betaine) directly affect DNA and histone methylases via their actions as precursors to SAM, and thereby influence epigenetic modifications [25,26,27,28,29]. Importantly, growing evidence has also emerged for the role of dietary methyl donors to impact gut microbiota composition [20,30,31].

While there is substantial evidence for maternal diet via its effects on one carbon transfer to influence long-term metabolism and disease risk in offspring [32], less is known about paternal diet. A nutritionally poor diet in fathers has been shown to affect cellular one-carbon metabolism by increasing levels of serum and tissue Hcy, which negatively affects male fertility [33]. Moreover, alterations in DNA methylation have been assessed in the sperm of infertile males in human [34] and animal [35] studies, where both showed impaired spermatogenesis, with the rodent model observing this impairment among F3 progeny as well. Interestingly, in sub-fertile men, folate supplementation can improve sperm quality [36]. 

Previous work showed that maternal diets supplemented with methyl donors attenuate adverse phenotypes in offspring associated with maternal high fat diet intake [37]. To our knowledge, this is the first study to examine whether a paternal diet supplemented with a methyl donor cocktail of betaine, choline, folic acid and vitamin B12 attenuates the adverse metabolic outcomes, epigenetic changes and gut microbial effects of a paternal high fat/sucrose diet in offspring.

## 2. Results

### 2.1. Paternal HF/S+M Decreases Adiposity in Adult Male and Female Offspring

**Figure 1:**
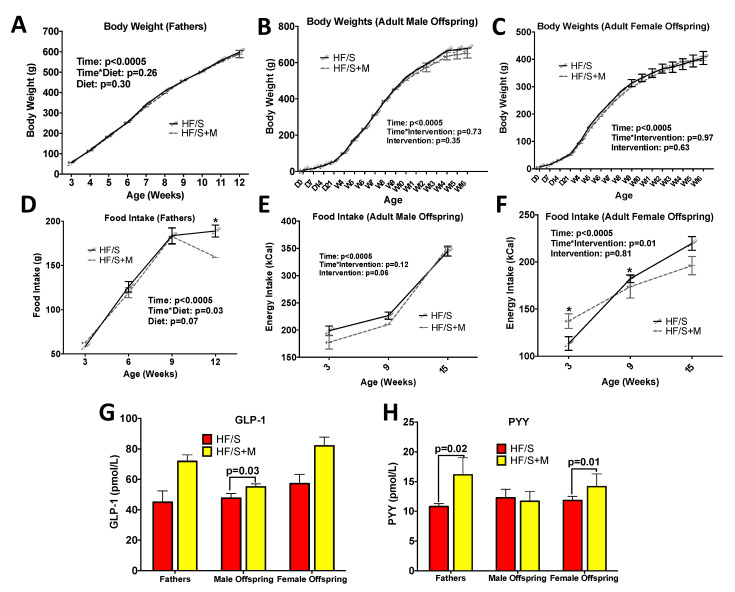
Body Weight, Food Intake and Gastrointestinal Peptides. The body weight of (**A**) fathers, (**B**) adult male offspring and (**C**) adult female offspring. The food intake of (**D**) fathers, (**E**) male offspring (**F**) female offspring; (**G**) serum glucagon-like peptide-1 (GLP-1) and (**H**) peptide tyrosine tyrosine (PYY)**.** The values are means ± SEM, *n* = 8–13. In adult offspring, there was a significant sex effect in the overall model for bodyweight (*p* = 0.0001), food intake (*p* = 0.0001), GLP-1 (*p* = 0.002); therefore, subsequent analysis was performed in males and females separately. * represents a significant difference between groups, *p* < 0.05.

In fathers, no difference in body weight (Figure 1A), body composition (Table 1) or relative organ weights was observed (Table 2) between HF/S and HF/S+M groups. From 4 weeks of age until euthanasia, offspring exhibited a significant sex effect (*p* < 0.05) for body weight, body composition and relative organ weight, therefore sexes were analyzed separately. No difference in body weight was observed between diets in male (Figure 1B) or female (Figure 1C) offspring; however, we observed important differences in body composition, wherein male and female HF/S+M offspring had significantly lower fat mass compared to HF/S offspring, even though both groups consumed the same, nutritionally complete, AIN-93 diet for 13 weeks (Table 1). Male bone mineral content was significantly reduced in HF/S+M compared to HF/S (Table 1). No differences were observed in organ weight as a percentage of body weight for the heart, liver, kidney, cecum or colon in male and female adult offspring or testes in male offspring (Supplementary Table S1). 

Energy intake was reduced in HF/S+M fathers compared to HF/S at 12 weeks of age (Figure 1D), which was similarly seen in female offspring at 9 weeks of age (Figure 1F). To examine hormonal influences on food intake, we assessed serum concentrations of glucagon-like peptide-1 (GLP-1) and peptide tyrosine tyrosine (PYY), both known to reduce food intake. GLP-1 was increased in adult HF/S+M male offspring compared to HF/S (*p* = 0.03) (Figure 1G). PYY was significantly increased in fathers consuming the HF/S+M diet (*p* = 0.02) (Figure 1H). Higher PYY was similarly seen in adult female offspring (*p* = 0.01) (Figure 1H).

### 2.2. Paternal HF/S+M Reduces Fasting Insulin and Insulin Resistance 

**Figure 2:**
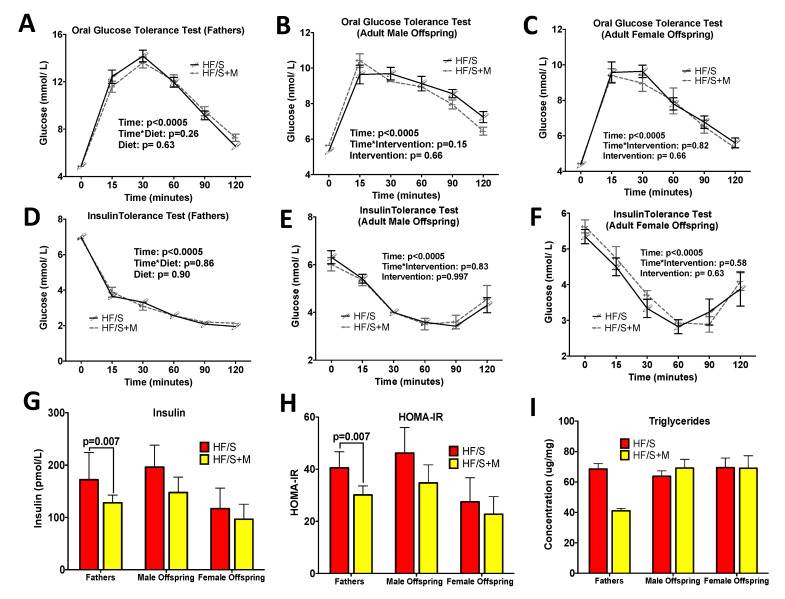
OGTT, ITT, Fasted Insulin, HOMA-IR and Hepatic Triglyceride Concentrations. The oral glucose tolerance test (OGTT) of (**A**) fathers, (**B**) adult male offspring and (**C**) adult female offspring. The insulin tolerance test (ITT) of (**D**) fathers, (**E**) male offspring, (**F**) female offspring. (**G**) Insulin levels, (**H**) Homeostatic Model Assessment of Insulin Resistance (HOMA-IR), (**I**) triglyceride content in hepatic tissue. The values are means ± SEM, *n* = 8–13. In adult offspring, there was a significant sex effect in the overall model for OGTT (*p* = 0.0001), ITT (*p* = 0.003), insulin (*p* = 0.0001), HOMA-IR (*p* = 0.0001). Triglyceride sex effect was not significant. Since most assessments in adult offspring had a significant sex effect, subsequent analysis was performed in males and females separately.

Next, we examined the possible influence of paternal methyl donor diet supplementation on offspring metabolic parameters. Blood glucose concentrations during the oral glucose tolerance test (OGTT) and insulin tolerance test (ITT) in fathers (Figure 2A,D), male (Figure 2B,E) and female (Figure 2C,F) offspring were not independently affected by diet or the interaction of diet and time. Prior to said investigations, a potential sex effect was assessed for glycemia during the OGTT and ITT. Both tests yielded significant sex effects (*p* < 0.05), therefore sexes were analyzed separately. Although no differences were seen in glycemia, we did identify a significant decrease in serum insulin (Figure 2G) and insulin resistance as assessed by the Homeostatic Model Assessment of Insulin Resistance (HOMA-IR) (Figure 2H) in HF/S+M fathers compared to HF/S. This was not evident in offspring. We did not find any differences in hepatic triglyceride concentrations in fathers or offspring, although, we did observe a 40% reduction of hepatic triglyceride concentration in HF/S+M fathers (41.0 ± 1.7 µg triglycerides/mg of liver tissue) compared to HFS fathers (68.7 ± 3.5 µg/mg) (Figure 2I).

### 2.3. HF/S+M Improved Markers of Reproductive Performance in Fathers

Since the effectiveness of maternal methyl supplementation on reproduction and pregnancy has been demonstrated [38], we examined whether similar effects may be observed following paternal methyl supplementation. We found no difference in the fertility index between groups, which was calculated as number of successful pregnancies divided by number of sperm positive fathers (Table 3). Notably, we found a significant difference in nights cohabited until conception, wherein HF/S+M resulted in fewer days to conception (Table 3). Similarly, we observed 300% fewer stillbirths in the HF/S+M group compared to HF/S group (Table 3).

### 2.4. Paternal HF/S+M Consumption Improved Epigenetic Markers in Fathers and Offspring

**Figure 3:**
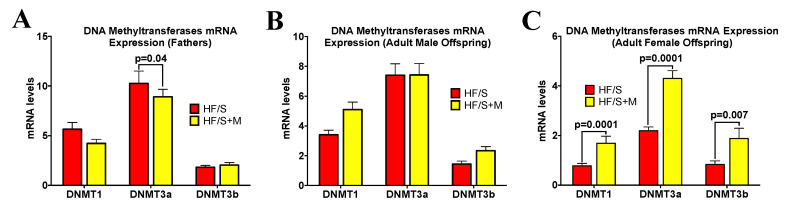
The adipose tissue mRNA levels of DNA Methyltransferases (DNMTs) in (**A**) fathers, (**B**) adult male offspring and (**C**) adult female offspring. The values are means ± SEM, *n* = 8–13. Sex differences were observed in DNMT1 (*p* = 0.0001), DNM3a (*p* = 0.0001) and DNMT3b (*p* = 0.03); therefore, subsequent analysis was performed in males and females separately.

Due to the fact that DNA methylation is a key epigenetic regulator of adipose tissue development and gene regulation [17], we examined DNA methyltransferases 1, 3a and 3b, considered important catalysts of DNA methylation [39]. Using RT-PCR, we found reduced expression of DNMT3a in retroperitoneal adipose tissue in fathers in the HF/S+M group (Figure 3A). Adult offspring DNMTs were significantly affected by sex (*p* < 0.001), therefore male and female offspring were assessed separately. In adult female offspring, we saw significantly elevated retroperitoneal adipose tissue expression of DNMT1, DNMT3a and DNMT3b in the HF/S+M group (Figure 3C).

Given that the differential expression of several miRNAs in the liver has been associated with obesity and insulin resistance [40], we assessed a panel of 10 microRNAs in liver tissue. We identified 4 out of 10 microRNAs that were differentially expressed in HF/S+M fathers; 3 were upregulated (miR-33, miR-103 and miR-107) and 1 was downregulated (miR-34a) (Table 3). In adult males, 4 microRNAs were differentially expressed; miR-24, miR-33, miR-122a, miR-143 were all upregulated in HF/S+M offspring (Table 3). In females, miR-33 was downregulated in HF/S+M versus HF/S offspring (Table 3).

### 2.5. Gut Microbiota and Short Chain Fatty Acids Are Impacted by Paternal HF/S+M Consumption Intergenerationally

**Figure 4:**
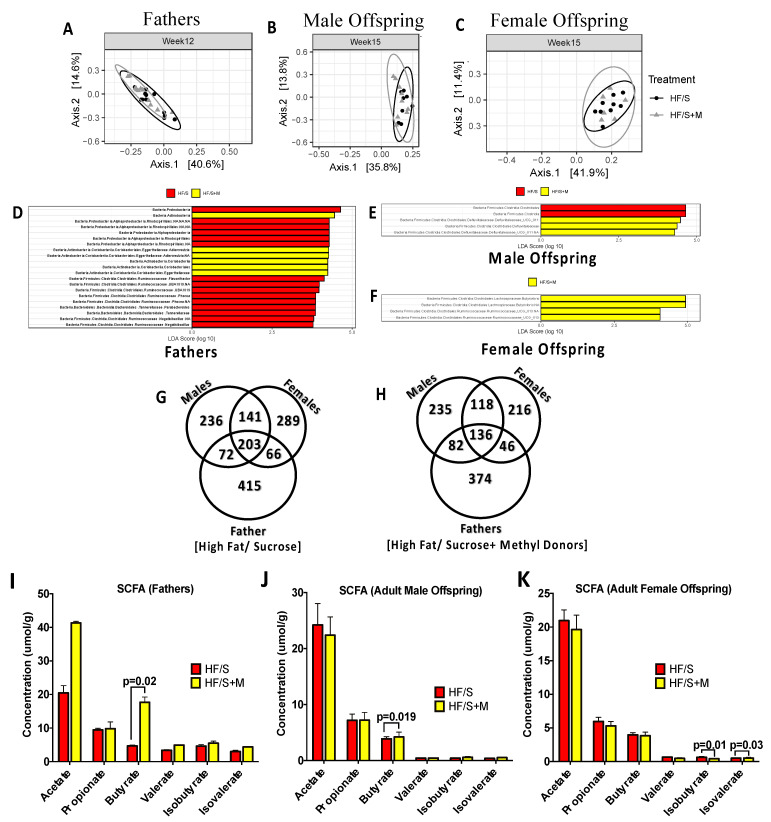
Fecal microbiota comparisons of fathers fed HF/S and HF/S+M and the intergenerational similarities in male and female offspring. Beta Diversity of (**A**) paternal at 12 weeks of age, (**B**) male offspring and (**C**) female offspring at 15 weeks of age, assessed with principal coordinates analysis (PCoA) using a Bray–Curtis distance matrix. Linear discriminant analysis effect size (LefSe) comparison of (**D**) Fathers at 12 weeks of age, (**E**) Male offspring and (**F**) Female offspring at 15 weeks of age. A Venn diagram comparison of ASVs that overlap between fathers and offspring and those only present in offspring, stratified by sex and (**G**) HF/S diet (**H**) HF/S+M diet. Cecal Short Chain Fatty Acids in: (**I**) paternal, (**J**) adult male offspring and (**K**) adult female offspring at euthanasia. Values are means ± SEM, *n* = 8–13 (*p* < 0.05).

Paternal methyl donor supplementation with HF/S diet impacted offspring fecal microbiota as seen by 16S rRNA sequencing of the V3 and V4 regions. There were no differences in alpha diversity at weaning in fathers or offspring (Supplementary Table S2), however, at 9 weeks of age, fathers supplemented with methyl donors showed significantly higher alpha diversity compared to the HF/S group as seen by Chao1 (*p* = 0.049) (Table 4). Interestingly, the opposite was observed in female adult offspring at 9 weeks of age, where HF/S+M group displayed reduced alpha diversity compared to HF/S group across all three alpha diversity indices (*p* < 0.01) (Table 4). None of these differences persisted to 12 or 15 weeks of age in fathers or offspring (Supplementary Table S3). Only a trend towards a difference in beta diversity in fathers at 12 weeks of age was observed (*p* = 0.055) (Figure 4A) while no differences were found in offspring at any age (Figure 4B,C). Linear discriminant analysis effect size (LEfSe) showed that fathers fed a HF/S+M diet had increased relative abundance of Actinobacteria, Adlercreutzia, Coriobacteriales, and *Eggerthellaceae* at 12 weeks of age compared to HF/S (Figure 4D). Adult HF/S male offspring at 15 weeks of age, showed an increased abundance of Clostridiales compared to HF/S+M (Figure 4E). HF/S+M males showed an increased abundance of *Defluviitalaceae* compared to HF/S (Figure 4E). Adult HF/S+M females exhibited an increased abundance of *Butyrivibrio* (Figure 4F).

The Venn diagrams show that paternal HF/S (Figure 4G) and HF/S+M (Figure 4H) groups have a total of 1422 and 1207 amplicon sequence variants (ASVs), respectively, at 12 and 15 weeks of age in fathers and offspring. Of those ASVs, the offspring shared 45% with the fathers in the HF/S group and 41% among HF/S+M.

Paternal HF/S intake supplemented with methyl donors altered cecal short chain fatty acid (SCFA) concentrations in fathers and offspring. Paternal HF/S+M significantly increased cecal butyrate (Figure 4I), which persisted in adult male offspring (Figure 4J). Adult female offspring showed significantly reduced isobutyrate and elevated isovalerate in the HF/S+M group compared to the HF/S group (Figure 4K).

## 3. Discussion

**Figure 5:**
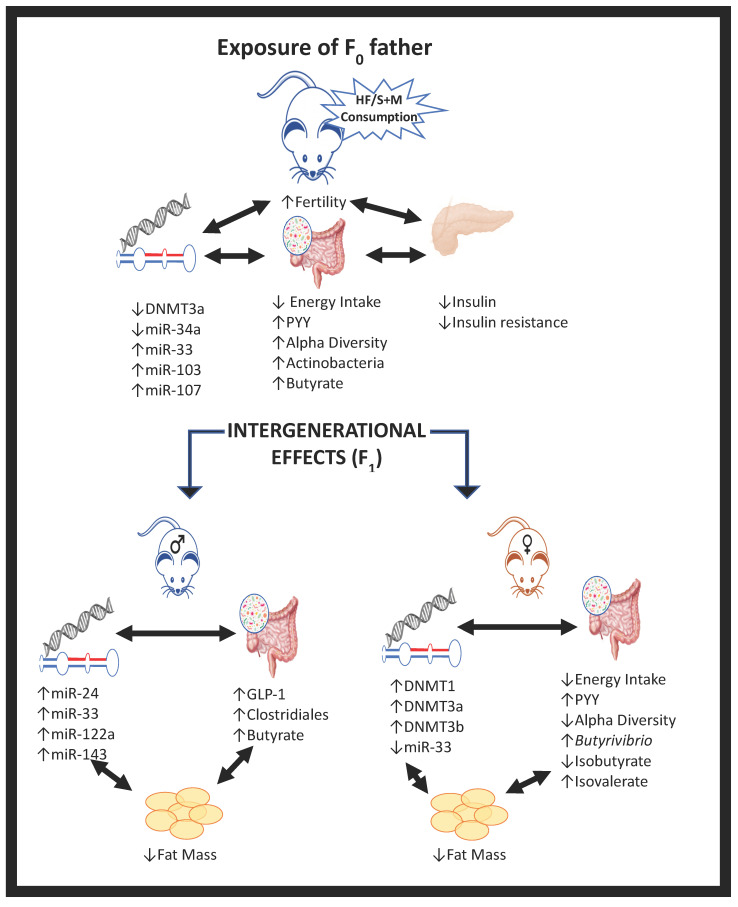
An overall summary of the major findings in fathers and adult offspring following a HFS diet, supplemented with a methyl donor cocktail of betaine, choline, folic acid and vitamin B12. All data are compared to a control HF/S diet.

We present evidence that supplementing a paternal HF/S diet with a methyl-donor cocktail of betaine, choline, folic acid and vitamin B12 before conception reduced energy intake, fasting serum insulin and insulin resistance alongside alterations in gut microbial signatures, epigenetic markers modulating metabolism, and reproductive outcomes in fathers. Paternal HF/S+M also appears to reduce fat mass and alter microRNA and gut microbial signatures in adult male and female offspring compared to HF/S intake alone (see Figure 5 for a summative schematic).

It has been postulated that epigenetic changes owing to malnutrition in utero have a substantial impact on transgenerational metabolic abnormalities [41]. Etiological studies have demonstrated that paternal BMI affected offspring BMI in a way that was independent of, but additive to, the BMI of the mother [42,43]. These findings in humans were furthered through a seminal study by Masuyama and colleagues in 2016 [44]. They examined whether high-fat diet-induced-obesity in fathers before conception would impact the metabolic status of offspring, as seen by the epigenetic status of the adiponectin and leptin gene promotors in a mouse model [44]. They also investigated whether a normal, control diet would reverse the epigenetic changes in subsequent generations [44]. In the F1 generation, epigenetic changes were diminished, whereas in the F2 generation, epigenetic changes caused by a paternal high fat diet were completely absent in male offspring [44]. Based on the reversal seen with a control diet, we sought to examine whether a HF/S diet supplemented with methyl donors could attenuate some of the detrimental metabolic outcomes caused by a pre-conception paternal HF/S diet in the F1 progeny. Our model uniquely shows that a paternal high fat diet supplemented with methyl-donors attenuates the accumulation of fat mass in adult male and female offspring. This was accompanied by changes in the expression of DNMTs and miRNAs, albeit differently according to sex.

Previous work has demonstrated that epigenetic changes, including DNA methylation play an important role in modulating gene expression [45,46] and have key roles in obesity-associated gene expression by governing transcriptional dysregulation [47,48]. One such transcriptional dysregulation occurs in the expression of genes involved in fat metabolism causing the decreased expression of adiponectin in adipose tissue of high-fat diet-induced obese models (DIOs) [49]. Given that the gut microbiota participates in epigenetic processes through its metabolites, such as folate and SCFAs, Yao et al. [49] investigated whether altering the gut microbiota with antibiotics affected the transcriptional expression of obesity-related genes such as adiponectin through epigenetic regulation. They showed that antibiotics given to DIO animals upregulated the expression of adiponectin in adipose tissue which was accompanied by a reduction in DNA methylation of the adiponectin promoter and the downregulation of DNMT1 and 3a [49]. Previous work from the same lab found that SCFA supplementation in a DIO model reversed the transcriptional alterations in adiponectin in adipose tissue, which was again mediated by reduced expression of DNMTs including DNMT3a [50]. This is consistent with our findings, wherein methyl-donor supplemented HF/S fathers showed reduced expression of DNMT3a in adipose tissue. Although, this was not accompanied by reductions in adiposity directly in the HFS+M fathers, we did see reductions in HFS+M offspring fat mass, suggesting a potential heritable influence of reduced DNMT3a expression in fathers and a subsequent reduction in adiposity in offspring. Although both male and female HFS+M offspring had reduced fat mass, it is interesting that female HF/S+M offspring showed increased expression of DNMTs including: 1, 3a and 3b, suggesting a sex-specific effect of one carbon metabolism. This might be explained by sex-specific disparities in epigenetic machinery like DNA methylation and histones that have been reported previously [51,52]. Alternatively, the sex difference observed may be due to the established difference in fat distribution and homeostasis between males and females [53], which may also alter the epigenetic machinery and function in adipose tissue, including DNA methylation.

The gut microbiota produces SCFAs which interact with the host epigenetic machinery, including DNA and histones, which are able to influence the host’s epigenetic state and function [54,55] including lipid metabolism and ultimately weight regulation. The gut microbiota synthesize choline, thiamin (vitamin B1), vitamin B2, nicotinic acid (vitamin B3), pantothenic acid (vitamin B5), pyridoxine (vitamin B6), biotin (vitamin B7), folate, and vitamin B12 [56,57]. Whether or not dietary supplementation with these methyl donors has the ability to correct some of the microbial dysbiosis observed with HF/S consumption is not well understood, particularly with regards to intergenerational effects. In HFS+M fathers, we saw increased relative abundance of *Adlercreutzia,* Coriobacteriales, and *Eggerthellaceae*. Coriobacteriales exert saccharolytic activity (fermentation of carbohydrates) in the gut [58], which could reflect the higher butyrate concentrations we detected in the cecal matter of HFS+M fathers and male offspring. Further, there has been a suggestion that members of the order Coriobacteriales may be indicators of a healthy gut microbiota community [59]. It is of interest that supplementing a HF/S diet with polyphenol-rich cranberry powder increased the relative abundance of both Coriobacteriales and *Eggerthellaceae* in mice [60]. *Eggerthellaceae*, which has been linked to positive effects in lipid metabolism, was also inversely correlated with body weight gain in mice [60]. Although relatively little is known about the genera *Adlercreutzia*, it has been shown in pubertal human subjects to be positively associated with testosterone [61]. *Adlercreutzia* have also been shown to metabolize phytoestrogens [62] and it is therefore possible that this bacteria could be affected by sex hormones, although this warrants further investigation.

Offspring gut microbiota did not differ as demonstrated by a principal coordinates analysis (PCoA) beta diversity assessment and little difference was observed at lower taxonomic levels as seen by LEfSe analysis. This might be indicative of the diminished metabolic influence of a paternal HF/S diet in the F1 progeny, as discussed in previous work [44]. Interestingly, however, males showed increased abundance of *Defluviitaleaceae*, which is reported to increase the statin efficacy of Rosuvastatin, a blood lipid-lowering agent in hyperlipidemia in humans [63]. Therefore, although very little is currently known about these bacteria, it is possible that the increased *Defluviitaleaceae* in male offspring could have contributed to altered lipid metabolism and reduced body fat mass.

MicroRNAs (miRNAs) are important post-transcriptional regulators of gene expression that have been implicated in pathways underpinning metabolic disease in multiple organs including the pancreas, liver, adipose tissue, and skeletal muscle [64]. Here, HFS+M fathers exhibited decreased hepatic expression of miR-34a and increased miR-103, miR-107 and miR-33. HFS+M female offspring similarly showed a trend towards decreased miR-34a in liver tissue (*p* = 0.09). MiR-34a is an important mediator in lipid homeostasis in the liver [65]. MiR-34 is characteristically elevated in the liver of DIO mice [66]. For instance, mice treated with anti-sense oligonucleotides that target miR-34a experienced improvements in glucose tolerance and insulin resistance, suggesting miR-34 to be a crucial target to mitigate insulin resistance. We showed that oral supplementation of a methyl-donor cocktail in our DIO rat model elicited similar results, wherein our HF/S+M fathers displayed improvements in insulin resistance. Additionally, Tryndyak and colleagues [67] showed that circulating miR-34a is the strongest correlate with non-alcoholic fatty liver disease-specific liver pathomorphology, wherein increased levels of plasma miR-34a increase overall liver pathology, as measured by total hepatic lesions and severity [67]. Although not reaching significance, we did observe a 40% decrease of triglyceride concentrations in the livers of HF/S+M fathers compared to HF/S.

MiR-103 and 107 have been previously identified as negative regulators of insulin sensitivity [68], and increased hepatic expression has been observed in both humans and murine models of metabolic disease and/or high fat diet consumption [68]. However, there are also studies suggesting that increasing miR-103 and miR-107 expression is beneficial. For example, obesity induced by a high-fat, high-cholesterol diet in mice decreased the expression of hepatic miR-103 and -107, while simultaneously increasing fatty acid synthase protein (FASN), a modulator of fatty acid synthesis [69]. FASN is a putative miR-107 target. Furthermore, Zhang et al. [70] showed that overexpression of miR-103 in mice fed a diet high in fructose and sucrose alleviated hepatic lipid accumulation and suppressed lipogenesis in the liver. We postulate that the increased expression of HF/S+M paternal hepatic miR-107 in our study could have reduced FASN, contributing to the observed reduction in fat mass in male and female adult offspring.

MiR-33 is yet another important regulator of lipid metabolism [71]. Inhibiting miR-33 function in vivo increases circulating high-density lipoprotein concentrations and lowers very-low-density lipoprotein and triglycerides by increasing the expression of fundamental enzymes involved in fatty acid oxidation [72]. Adult female offspring of HF/S+M fathers exhibited reduced expression of miR-33, potentially explaining the concurrent reduction in adiposity in females. Additionally, miR-33 plays an important role in regulating insulin signaling by targeting insulin receptor substrate 2, a vital component of insulin signaling in the liver [73]. Conversely, miR-33-knockout in mice showed deleterious outcomes, including increased obesity, insulin resistance and food intake [74]. This study elicited a miR-33 conundrum, which was similarly observed in our study. Methyl donor supplementation increased hepatic miR-33 expression in fathers and adult male offspring but reduced it in females. In HFS+M fathers, reduced miR-33 expression occurred alongside reduced insulin resistance, while in HFS+M offspring the disparate expression was associated with reduced adiposity in both male and female HFS+M offspring. We also observed reduced energy intake in HFS+M fathers at 12 weeks of age and 9 weeks of age in female offspring, which could be attributed in part to increased levels of appetite regulating gut hormones like PYY. It is worth noting, with the exception of miR-33 in fathers and adult male offspring, we observed differentially acquired microRNAs intergenerationally. This may be due to the fact that the epididymis, containing mature sperm, facilitates altered microRNA transfer enacted by epididymosomes [75]. Future research should assess this epididymis-specific microRNA alteration in vivo.

In male HFS+M offspring, we saw a substantial increase in miR-122a. Benatti et al. [76] showed that maternal high-fat diet consumption modulates hepatic lipid metabolism and microRNA expression in offspring, most notably showing reductions in miR-122a in DIO mice. They concluded that a maternal high fat diet impairs offspring lipid metabolism and miRNA expression, which may have lasting metabolic impairments in adulthood [76]. We found that paternal HF/S supplemented with methyl donors ameliorated these effects and in turn increased miR-122a in male offspring.

To our knowledge, no human studies have been conducted that examine whether paternal methyl-donor supplementation could mitigate some of the detrimental reproductive and metabolic effects of a paternal HF/S diet. Given the increasing recognition of the importance of paternal health and nutritional intake on programming metabolism in offspring, future work is warranted to determine the potential for individual methyl donors or cocktails such as we have used here to positively affect male fertility and pregnancy outcomes in humans.

## 4. Materials and Methods

### 4.1. Animal Model and Dietary Treatment

Maintained in a temperature and humidity-controlled facility, twenty-four male Sprague Dawley rats (Charles River Laboratories, Montreal, QC, Canada) were randomized to one of two dietary interventions: (1) high fat/high sucrose (HF/S) or (2) high fat/high sucrose supplemented with a methyl donor cocktail (HF/S+M) [betaine (5 g/kg diet), choline (5.37 g/kg diet), folic acid (5.5 mg/kg diet), vitamin B12 (0.5 mg g/kg diet); (Sigma Aldrich, Oakville, ON, Canada) according to previous work [77,78,79]. HF/S diets were purchased from Dyets Inc. (Bethlehem, PA, USA) (DYETS# 103915: age 3–9 weeks; DYETS# 102412: weeks 10–12). Diet composition is provided in Supplementary Table S4. At 12 weeks of age, a virgin female Sprague Dawley rat was co-housed with a male rat from one of the dietary interventions during the dark cycle for as many consecutive nights until a copulation plug was identified. During the light cycle, females were given an American Institute of Nutrition (AIN)-93G diet and males were returned to their designated dietary intervention with water ad libitum. During pregnancy and lactation, dams consumed an AIN-93G diet. In an effort to limit differences in energy intake due to variances in litter size, one day after birth, litters were culled to 10 offspring (*n* = 5 males; *n* = 5 females). Litters that were less than *n* = 10 were increased via cross-fostering with offspring from another litter belonging to the same treatment group. At 3 weeks of age, one male and one female from each litter (considered as *n* = 1) were weaned onto AIN-93G diet (weeks 3–9) and AIN-93M (weeks 10–12) and water ad libitum for 13 weeks. This study was approved by the University of Calgary Animal Care Committee (AC18-0074) and conformed to the *Guide to the Care and Use of Laboratory Animals*.

### 4.2. Body Weight, Food Intake and Body Composition

Throughout the duration of the study, paternal and offspring bodyweights were quantified weekly; food intake was quantified every 3 weeks. A Dual X-ray Absorptiometry (DXA) scan (Hologic ODR 4500; Hologic Inc., Marlborough, MA, USA) was used to assess body composition 1 day prior to sacrifice. To ensure animals remained still during the scan, animals were lightly anaesthetized using isoflurane. Using QDR software for small animals, bone mineral content/density (BMC/BMD) (g and g/cm^2^), fat mass (g), lean mass (g) and body fat % were quantified.

### 4.3. Oral Glucose Tolerance Test (OGTT) and Insulin Tolerance Test (ITT)

At 10 and 14 weeks of age, in fathers and offspring, respectively, rats underwent a 12 h fast and a blood glucose measurement was obtained via tail nick and a One Touch Ultra^®^ 2 glucose meter (Lifespan, Burnaby, BC, Canada), accounting for the 0 min timepoint. Additional blood glucose measurements were collected at 15, 30, 60, 90 and 120 min after a 2 g/kg glucose solution was administered via oral gavage.

In fathers and offspring at 11 and 15 weeks of age respectively, rats were fasted for 6 h and blood glucose measured via tail nick using a One Touch Ultra^®^ 2 glucose meter (Lifespan, Burnaby, Canada) accounting for the 0 min timepoint. Additional blood glucose measurements were collected at 15, 30, 60, 90 and 120 min after an intraperitoneal injection of insulin (0.75 U/kg).

### 4.4. Tissue Harvest and Blood Insulin, GLP-1, PYY and HOMA-IR

The animals were anesthetized using isoflurane and denied access to food overnight for a 12 h fast; 1 mL of blood was collected from the portal vein in a chilled tube containing diprotinin-A (0.034 mg/mL blood; MP Biomedicals, Irvine, CA, USA), Sigma protease inhibitor (1 mg/mL blood; Sigma Aldrich, Oakville, ON, Canada) and Roche Pefabloc (1 mg/mL of blood; Roche, Mississauga, ON, Canada). Plasma was collected after centrifugation and stored in −80 °C until insulin, peptide tyrosine tyrosine (PYY) and glucagon-like peptide 1 (GLP-1) were measured using a Rat Metabolic Multiplex Array (MRDMET) (Millipore, St. Charles, MO, USA) (Eve Technologies, Calgary, AB, Canada). The animals were henceforth euthanized via decapitation and heart, liver, kidney, cecum, colon and male testes were weighed and stored in −80 °C until analysis. The Homeostatic Model Assessment of Insulin Resistance (HOMA-IR) was used to estimate insulin resistance using the following formula [80]:

HOMA-IR = [glucose (mmol/L) × insulin (mIU/mL)]/22.5(1)

### 4.5. Hepatic Triglyceride Analysis

Triglyceride concentrations were assessed from a starting amount of 25 mg of liver derived from the right lobe, using the GPO reagent set according to manufacturer’s instructions (Pointe Scientific Inc., Lincoln Park, MI, USA).

### 4.6. Gut Microbiota 16S rRNA Gene Sequencing

Baseline fecal matter was collected at 3 weeks of age in fathers and offspring. Additional fecal matter was collected at 9 and 12 weeks of age in fathers and 9 and 15 weeks of age in offspring. All fecal matter was snap frozen and stored at −80 °C until analysis. Gut microbial 16S rRNA gene sequencing was performed according to our previous work [81,82]. Briefly, a FastDNA spin kit for feces (MP Biomedicals, Lachine, QC, Canada) was used to extract bacterial DNA according to manufacturer’s guidelines. Bacterial DNA concentrations were diluted to 4 ng/uL. The MiSeq Illumina platform was utilized to amplify the V3 and V4 region of the 16S rRNA gene (Illumina, San Diego, CA, USA) at the Centre for Health Genomics and Informatics (University of Calgary, Calgary, AB, Canada).

### 4.7. Cecal Short Chain Fatty Acids

SCFAs were extracted from cecal matter and assessed using reverse-phase HPLC on a c18 column as previously described [83].

### 4.8. RT-PCR of DNA Methyltransferase mRNA and microRNAs

Total RNA was extracted from retroperitoneal adipose tissue using the RNAeasy Lipid Tissue Mini Kit (Qiagen) and then reverse-transcribed into cDNA using the SuperScript II RT (Qiagen). RT-PCR was performed as previously described [84]. The mRNA expression of DNA methyltransferase (DNMT) 1, 3a and 3b of all samples were analyzed relative to the 18S housekeeping control gene using the 2^−ΔCT^ method [85]. The amplicon context sequences are provided in Supplementary Table S5.

Using the miRNeasy mini kit (Qiagen), following the manufacturer’s instructions for Purification of Total RNA, including Small RNA from animal tissue, microRNAs were isolated from liver tissue and then reverse transcribed into cDNA using the miScript II RT kit (Qiagen). RT-PCR was conducted as previously described [84], wherein all microRNAs were analyzed relative to SNORD68 and SNORD96A controls genes using the 2^−ΔCT^ method [85]. MicroRNA primer sequences are listed in Supplementary Table S6.

### 4.9. Statistical and Bioinformatics Analysis

Statistical comparisons for all outcomes, except 16S rRNA sequencing data, were performed using IBM^®^ SPSS Statistics, version 24.0. A multivariate general linear model (GLM) was used to determine a sex effect between male and female offspring. If a sex effect was identified, males and females were analyzed separately using an independent samples t-test. Outcomes with multiple time points were analyzed using a repeated measures GLM, wherein diet was the between-subject factor and time was the within-subject factor. Identification of a significant interaction between diet and time was followed with an independent samples *t*-test to determine differences between dietary groups. All data were presented as mean± standard error of the mean (SEM).

Sequence data were first quality filtered using the filterAndTrim, assignTaxonomy and assignSpecies functions with the R package dada2 (version 1.10.1) [86]. Diversity analysis was conducted using R package phyloseq (version 1.24.2) [87], where alpha diversity was determined using ANOVA and Tukey HSD if significant. Beta diversity was assessed using PCoA (principal coordinates analysis) on a matrix of Bray–Curtis distances. A permutational multivariate analysis of variance (PERMANOVA) was performed to determine significant differences between dietary interventions. Differentially abundant features were assessed using a LEfSe analysis [88], using a significance of alpha = 0.05 and default parameters. Significance for all outcomes was set at *p* ≤ 0.05, unless stated otherwise.

## Figures and Tables

**Table 1:** The body composition of fathers at mating at 12 weeks of age and offspring at 16 weeks of age.

	HF/S	HF/S+M	*p*-Value
**Fathers**			
BMC (g)	15.82 ± 0.45	15.39 ± 0.43	0.85
BMD (g/cm^2^)	0.17 ± 0.002	0.17 ± 0.002	0.88
Fat Mass (g)	143.82.4 ± 11.53	123.0 ± 11.81	0.86
Lean+ BMC (g)	507.76 ± 13.84	491.72 ± 12.81	0.92
% Body Fat	21.82 ± 1.22	19.63 ± 1.37	0.68
**Male Offspring**			
BMC (g)	17.07 ± 0.18	16.67 ± 0.49	0.02
BMD (g/cm^2^)	0.18 ± 0.002	0.17 ± 0.002	0.34
Fat Mass (g)	135.11 ± 5.95	116.14 ± 1.77	0.005
Lean+ BMC (g)	526.17 ± 9.88	546.88 ± 14.48	0.12
% Body Fat	19.93 ± 0.73	16.83 ± 0.84	0.56
**Female Offspring**			
BMC (g)	11.78 ± 0.53	11.74 ± 0.41	0.19
BMD (g/cm^2^)	0.163 ± 0.002	0.164 ± 0.003	0.96
Fat Mass (g)	100.84 ± 18.78	95.53 ± 5.69	0.005
Lean+ BMC (g)	304.36 ± 9.29	293.33 ± 10.26	0.86
% Body Fat	23.54 ± 3.40	24.48 ± 0.88	0.10

**Table 2:** Reproductive markers for paternal fertility.

	HF/S	HF/S+M
Males (n)	13	12
Males cohabited with females	13	12
Nights cohabited until conception	3.00 ± 0.56	2.00 ± 0.33 *
# of successful pregnancies	10	9
Fertility Index (%)	77	75
# of pups born alive (per father)	14.7 ± 0.6	13.3 ± 0.9
# of stillbirth pups	3	1
Pup survival (%)	97.8	99.9
Relative abundance of male pups (%)	51.2 ± 4.1	49.2 ± 1.0
Relative abundance of female pups (%)	48.8 ± 4.1	46.98 ± 2.0

* *p* < 0.05.

**Table 3:** MicroRNA expression in liver tissue.

	HF/S	HF/S+M	*p*-Value
**Fathers**			
miR-21	472.0 ± 151.2	701.5 ± 244.9	0.78
miR-24	0.05 ± 0.01	0.05 ± 0.01	0.67
miR-33	0.00017 ± 0.00002	0.0002 ± 0.00006	0.03
miR-34a	1.02 ± 0.17	0.56 ± 0.06	0.002
miR-103	20.35 ± 1.91	21.65 ± 3.94	0.03
miR-107	0.32 ± 0.01	0.42 ± 0.01	0.04
miR-122a	5966.08 ± 1122.78	5822.70 ± 1310.41	0.51
miR-130a	0.02 ± 0.01	0.01 ± 0.01	0.10
miR-143	0.04 ± 0.01	0.03 ± 0.01	0.70
miR-let-7c	3.82 ± 0.89	5.05 ± 0.95	0.90
**Male Offspring**			
miR-21	1591.20 ± 722.13	1539.55 ± 476.72	0.47
miR-24	0.12 ± 0.02	0.15 ± 0.04	0.03
miR-33	0.00027 ± 0.00004	0.00034 ± 0.0009	0.006
miR-34a	0.66 ± 0.15	0.57 ± 0.10	0.36
miR-103	17.78 ± 2.70	17.57 ± 3.29	0.44
miR-107	0.34 ± 0.05	0.33 ± 0.03	0.16
miR-122a	8370.09 ± 1788.12	18,602.11 ± 5733.86	0.001
miR-130a	0.02 ± 0.004	0.01 ± 0.003	0.16
miR-143	0.03 ± 0.01	0.04 ± 0.002	0.009
miR-let-7c	6.15 ± 2.79	7.00 ± 3.02	0.60
**Female Offspring**			
miR-21	910.20 ± 221.59	723.52 ± 468.95	0.14
miR-24	0.07 ± 0.02	0.07 ± 0.01	0.42
miR-33	0.0002 ± 0.0001	0.0001 ± 0.00001	0.0001
miR-34a	1.07 ± 0.35	0.99 ± 0.16	0.09
miR-103	11.65 ± 1.00	15.47 ± 2.16	0.09
miR-107	0.57 ± 0.11	0.58 ± 0.02	0.11
miR-122a	12,872.52 ± 4519.43	8144.62 ± 1220.41	0.21
miR-130a	0.007 ± 0.002	0.01 ± 0.003	0.16
miR-143	0.02 ± 0.006	0.02 ± 0.002	0.16
miR-let-7c	12.86 ± 3.90	6.2 ± 0.64	0.06

**Table 4:** Alpha diversity for fathers and offspring at 9 weeks of age.

	HF/S	HF/S+M	*p*-Value
**Paternal**			
Chao1	209.19 ± 8.37	265.78 ± 26.87	0.049
Shannon	3.87 ± 0.09	3.95 ± 0.13	0.61
Simpson	0.95 ± 0.004	0.95 ± 0.01	0.99
**Male Offspring**			
Chao1	182.82 ± 28.3	168.67 ± 7.09	0.63
Shannon	3.76 ± 0.08	3.83 ± 0.08	0.54
Simpson	0.95 ± 0.01	0.95 ± 0.01	0.55
**Female Offspring**			
Chao1	192.31 ± 11.22	139.44 ± 11.48	0.004
Shannon	3.99 ± 0.06	3.67 ± 0.07	0.002
Simpson	0.96 ± 0.003	0.95 ± 0.004	0.004

The values are means ± SEM, *n* = 8–13.

## Data Availability

Data is available from the corresponding author upon reasonable request.

## Supplementary Materials

Supplementary Materials can be found at https://www.mdpi.com/1422-0067/22/2/689/s1.

## Abbreviations

DNMT	DNA methyltransferase
HF/S	High fat/high sucrose
HF/S+M	High fat/high sucrose supplemented with methyl donor cocktail
Hcy	Homocysteine
HOMA-IR	Homeostatic Model Assessment of Insulin Resistance
miR	microRNA
ncRNAs	Non-coding RNAs
SAM	S-adenosylmethionine
SCFAs	Short chain fatty acids
