# Supplementary material for: Paternal Methyl Donor Supplementation in Rats Improves Fertility, Physiological Outcomes, Gut Microbial Signatures and Epigenetic Markers Altered by High Fat/High Sucrose Diet
Source: Int J Mol Sci. 2021 Jan 12;22(2):689. doi: 10.3390/ijms22020689 (PMC7826956; doi:10.3390/ijms22020689)
Supplement: Supplementary file 1 [file ijms-22-00689-s001.pdf]

Table S1 Organ Weights of Fathers and Adult Offspring at euthanasia

	HF/S	HF/S+M	p-value
**Fathers**			
Body Weight (g)	651.6±21.2	614.7±21.9	0.20
Organ weight (% BW)			
Heart	0.27±0.01	0.28±0.01	0.38
Liver	3.00±0.08	2.99±0.08	0.75
Kidney	0.29±0.01	0.29±0.01	0.26
Cecum	0.09±0.003	0.10±0.004	0.70
Colon	0.21±0.01	0.22±0.01	0.78
Testes	0.29±0.01	0.29±0.01	0.86
**Male Offspring**			
Body Weight (g)	666.9±6.2	672±22.7	0.20
Organ weight (% BW)			
Heart	0.28±0.01	0.27±0.01	0.30
Liver	2.70±0.07	2.67±0.04	0.90
Kidney	0.252±0.005	0.251±0.008	0.07
Cecum	0.09±0.003	0.11±0.004	0.09
Colon	0.17±0.01	0.19±0.004	0.91
Testes	0.30±0.01	0.28±0.01	0.58
**Female Offspring**			
Body Weight (g)	405.2±21.2	388.8±14.3	0.40
Organ weight (% BW)			
Heart	0.30±0.01	0.30±0.01	0.29
Liver	2.59±0.05	2.79±0.12	0.07
Kidney	0.26±0.01	0.26±0.01	0.47
Cecum	0.12±0.01	0.11±0.01	0.75
Colon	0.20±0.01	0.13±0.01	0.41

Values are means ± SEM, *n* = 8-13. Male offspring heart and female kidney were log transformed for statistical analysis.

Table S2 Alpha Diversity at 3 Weeks of Age

	HF/S	HF/S+M	p-value
**Paternal**			
Chao1	269.77±11.47	257.04±13.85	0.49
Shannon	3.96±0.07	3.85±0.12	0.46
Simpson	0.95±0.004	0.94±0.01	0.47
**Male Offspring**			
Chao1	123.75±9.16	150.53±19.24	0.25
Shannon	3.77±0.05	3.83±0.08	0.50
Simpson	0.96±0.003	0.96±0.004	0.86
**Female Offspring**			
Chao1	179.27±20.31	173.33±11.55	0.80
Shannon	3.78±0.06	3.72±0.06	0.56
Simpson	0.95±0.003	0.95±0.004	0.29

Values are means ± SEM, *n* = 8-13.

Table S3 Alpha Diversity- Week 12 for Fathers and Week 15 for offspring

	HF/S	HF/S+M	p-value
**Paternal**			
Chao1	191.48±23.52	153.09±21.42	0.24
Shannon	4.00±0.1	3.77±0.11	0.14
Simpson	0.96±0.003	0.95±0.004	0.11
**Male Offspring**			
Chao1	140.53±8.79	153.39±16.61	0.52
Shannon	3.89±0.09	3.73±0.14	0.36
Simpson	0.96±0.004	0.94±0.01	0.20
**Female Offspring**			
Chao1	148.47±23.18	154.35±16.67	0.86
Shannon	3.64±0.13	3.64±0.17	0.98
Simpson	0.95±0.01	0.94±0.01	0.55

Values are means ± SEM, *n* = 8-13.

Table S4: Experimental diet composition from weeks 3-9 and 10-16

	HF/S	HF/S+M	HF/S	HF/S+M
**g/kg**	**Weeks 3-9**		**Weeks 10-16**	
**Casein**	240	237.5	200	197.9
**Sucrose**	459.5	454.7	499.5	494.3
**Soybean Oil**	100	99	100	99
**Lard**	100	99	100	99
**Alphacel**	50	49.5	50	49.5
**AIN-93M Mineral Mix**	35	34.6	35	34.6
**AIN-93 VX Vitamin Mix**	10	9.9	10	9.9
**DL-Methionine**	3	2.97	3	2.97
**Choline-Bitartrate**	2.5	2.47	2.5	2.47
**Betaine**	0	5	0	5
**Choline (CDP choline)**	0	5.37	0	5.37
**Folic Acid**	0	0.0055	0	0.0055
**Vitamin B12**	0	0.0005	0	0.0005
**Energy density (kJ/g)**	19.3	19.1	19.3	19.1
**Carbohydrate (% of kcal)**	49.8	49.3	49.8	49.3
**Protein (% of kcal)**	11.1	11.0	11.1	11.0
**Fat (% of kcal)**	39.1	38.7	39.1	38.7

The digestible energy of high fat/ sucrose diets was 4.58 kcal/g and 4.6 kcal/g for the 3-9 and 10-16 week formulations respectively. Diets were purchased from Dyets, Inc. (Bethlehem, PA, USA). Methyl donors (Betaine, Choline, Folic Acid and Vitamin B12) were purchased from Sigma Aldrich (Oakville, ON, Canada).

Table S5 Amplicon Context Sequence for DNMTs in adipose tissue

Gene	Amplicon Context Sequence
**Adipose Tissue**	
DNMT1	ATCGTCCTTAGCGTCGTCGTAACTTTCTACCTGGCTCACCA CAAACTGGGCATGGCGTAGGAGGGAGTCCTCGGTGAACCGG TTCACATTGATGGCAGAAGAGGAA CAGTGGTCTCGATCTTA TTGATCAAG
DNMT3a	GAATGATAAGCTGGAGTTGCAAGAGTGTCTGGAACACGGC AGAATAGCCAGTTCAGCAAAGTGAGGACCATTACCACCAG GTCAAACTCCATA
DNMT3b	TTCAGGCAGTAGGAACTTAGAAGCCAGGAGACGCGAGAACA AAAGTCGAGAC GCACAACCATTGACTTTGCCGCTTCTGAGTA CTCCACACCCC
**18S**	**Forward (5′→3′) TGACTCAACACGGGAAACC**
	**Reverse (3′→5′) TCGCTCCACCAACTAAGAAC**

18S was used as the housekeeping gene.

Table S6 Universal primer sequences for microRNA in liver tissue

microRNA	Universal Primer
**Liver**	
Rn_miR-21_2	CTAGCTTATCAGACTGATGTTG
Rn_miR-24_1	TGGCTCAGTTCAGCAGGAAC
Rn_miR-33_2	GTGCATTGTAGTTGCATTGCA
Rn_miR-34a_1	TGGCAGTGTCTTAGCTGGTTG
Rn_miR-103_2	AGCAGCATTGTACAGGGCTATG
Rn_miR-107_2	AGCAGCATTGTACAGGGCTATC
Rn_miR-122a_1	TGGAGTGTGACAATGGTGTTT
Rn_miR-130a_1	CAGTGCAATGTTAAAAGGGC
Rn_miR-143_1	TGAGATGAAGCACTGTAGCT
**Hs_SNORD68_11**	**TTTGAACCCTTTTCCATCTG**
**Hs_SNORD96A_11**	**GACATGTCCTGCAATTCTGAA**

Hs_SNORD68_11 and Hs_SNORD96A_11 were used as controls.
